# mTORC2 Deficiency Alters the Metabolic Profile of Conventional Dendritic Cells

**DOI:** 10.3389/fimmu.2019.01451

**Published:** 2019-07-02

**Authors:** Alicia R. Watson, Helong Dai, Yawen Zheng, Ryosuke Nakano, Anastasios D. Giannou, Ashley V. Menk, Donna B. Stolz, Greg M. Delgoffe, Angus W. Thomson

**Affiliations:** ^1^Department of Surgery, Starzl Transplantation Institute, University of Pittsburgh School of Medicine, Pittsburgh, PA, United States; ^2^Department of Pathology, University of Pittsburgh School of Medicine, Pittsburgh, PA, United States; ^3^Department of Urological Organ Transplantation, The Second Xiangya Hospital of Central South University, Changsha, China; ^4^Section of Molecular Immunology and Gastroenterology, Department of Medicine, University Medical Center Hamburg-Eppendorf, Hamburg, Germany; ^5^Tumor Microenvironment Center, University of Pittsburgh Cancer Institute, Pittsburgh, PA, United States; ^6^Department of Cell Biology, University of Pittsburgh, Pittsburgh, PA, United States; ^7^Department of Immunology, University of Pittsburgh School of Medicine, Pittsburgh, PA, United States

**Keywords:** mammalian target of rapamycin complex 2, rapamycin, dendritic cells, metabolism, mitochondrial regulation, mouse

## Abstract

In myeloid dendritic cells (DC), deletion of the mechanistic target of rapamycin complex 2 (TORC2) results in an augmented pro-inflammatory phenotype and T cell stimulatory activity; however, the underlying mechanism has not been resolved. Here, we demonstrate that mouse bone marrow-derived TORC2-deficient myeloid DC (TORC2^−/−^ DC) utilize an altered metabolic program, characterized by enhanced baseline glycolytic function compared to wild-type WT control (Ctrl) DC, increased dependence on glycolytic ATP production, elevated lipid content and higher viability following stimulation with LPS. In addition, TORC2^−/−^ DC display an increased spare respiratory capacity (SRC) compared to WT Ctrl DC; this metabolic phenotype corresponds with increased mitochondrial mass and mean mitochondrial DNA copy number, and failure of TORC2^−/−^ DC mitochondria to depolarize following LPS stimulation. Our data suggest that the enhanced metabolic activity of TORC2^−/−^ DC may be due to compensatory TORC1 pathway activity, namely increased expression of multiple genes upstream of Akt/TORC1 activity, including the integrin alpha IIb, protein tyrosine kinase 2/focal adhesion kinase, IL-7R and Janus kinase 1(JAK1), and the activation of downstream targets of TORC1, including p70S6K, eukaryotic translation initiation factor 4E binding protein 1 (4EBP1) and CD36 (fatty acid translocase). These enhanced TORC1 pathway activities may culminate in increased expression of the nuclear receptor peroxisome proliferator-activated receptor γ (Pparγ) that regulates fatty acid storage, and the transcription factor sterol regulatory element-binding transcription factor 1 (Srebf1). Taken together, our data suggest that TORC2 may function to restrain TORC1-driven metabolic activity and mitochondrial regulation in myeloid DC.

## Introduction

The mechanistic target of rapamycin (mTOR) is an integrative serine/threonine kinase in the PI3K family. In response to environmental cues, mTOR regulates cell growth/proliferation, and metabolism (1, 2), and immune cell function (3, 4). mTOR is known to function in two discrete complexes: mTOR complex 1 (mTORC1) (5) and mTORC2 (6). Assembled mTORC1 phosphorylates and activates the translational proteins ribosomal S6 kinase β-1 (S6K1) and eukaryotic translation initiation factor 4E-binding protein 1 (4E-BP1), and regulates cellular processes in a nutrient-dependent fashion (7). Conversely, mTORC2 is known to phosphorylate and activate Akt (protein kinase B), protein kinase C (PKC) and serum and glucocorticoid-regulated kinase 1 (SGK1) and to regulate actin cytoskeletal dynamics in fibroblasts (6).

The function of mTORC1 in dendritic cells (DC) has been studied extensively using the immunosuppressive pro-drug rapamycin (RAPA) (8–10). RAPA inhibition of mTORC1 in DC prevents their maturation, leading to decreased T effector cell proliferation and increased regulatory T cell (T_reg_) differentiation (8, 11, 12). While little had been known previously about the function of RAPA-insensitive mTORC2 (referred to subsequently as TORC2) specifically in DC, we have shown recently that functional TORC2 deletion specifically in these antigen-presenting cells (APC) leads to both an enhanced pro-inflammatory DC phenotype and Th1/Th17 allogeneic T cell polarization and proliferation (13). Additionally, intratumoral delivery of TORC2-deficient DC delays melanoma progression in a CD8^+^ T cell-dependent manner (14), whereas skin grafts from donors lacking TORC2 in DC undergo enhanced CD8^+^ T cell-mediated rejection (15). However, the mechanisms underlying these enhanced DC functions remain undefined.

There has been growing interest in defining the distinct roles that mTOR signaling plays in cell growth and metabolism (1, 16–18) and in the roles that TORC1 and TORC2 play in linking metabolic programming to cell activation, function and survival, both in T cell subsets (19–24) and more recently, in macrophages (25, 26), and DC (27–29). Like resting T cells, quiescent DC have relatively low metabolic needs; upon activation, however, bioenergetic demand increases to support upregulated co-stimulatory molecule expression and cytokine production (30, 31). Bone marrow (BM)-derived DC activated through Toll-like receptors (TLRs) meet this enhanced anabolic demand by increasing their dependence on aerobic glycolysis, as opposed to mitochondrial oxidative phosphorylation; glycolytic commitment is crucial for the survival of TLR-activated DC and is dependent on the PI3K/TORC1 signaling axis driving expression of hypoxia-inducible factor (Hif)-1α. Hif-1α induces increased glucose transporter expression, thereby biasing the cell toward glycolysis when NO production resulting from inducible NO synthase (iNOS) expression competes with oxygen for cytochrome oxidase at the mtiochondrial membrane (32). Indeed, inhibiting glycolysis in TLR-activated DC results in diminished co-stimulatory molecule expression and cytokine production (33).

These previous studies concluded that TORC2 did not play a role in regulating DC immunometabolism (34). However, the investigations were conducted using adenosine triphosphate (ATP)-competitive dual TORC inhibitors; therefore, the discrete function of TORC2 in linking metabolic programming and immune function in DC remains unclear. In addition, a recent study of tissue-resident peritoneal macrophages (26) demonstrated that TORC2 deficiency promoted their generation, and that TORC2-deficient macrophages and peritoneal resident macrophages had enhanced mitochondrial biomass, as well as an altered metabolic profile, compared to monocyte-derived macrophages.

In the present investigation, we have utilized DC generated from mice in which Rictor (an essential component for TORC2 assembly) is deleted specifically in CD11c^+^ DC (35) to ascertain whether the augmented inflammatory phenotype of TORC2^−/−^ DC is a consequence of altered cellular metabolism. We demonstrate that TORC2^−/−^ DC are biased toward glycolytic metabolism, have an increased dependence on glycolysis to generate ATP, elevated lipid content and higher viability following TLR4 agonism with bacterial lipopolysaccharide (LPS). In addition, TORC2^−/−^ DC have augmented spare respiratory capacity (SRC), mitochondrial mass and mean DNA copy number, and mitochondria that fail to depolarize following TLR agonism, as well as differential Golgi apparatus dispersal compared to WT control (Ctrl) DC. We also show that TORC1 inhibition by rapamycin in TORC2^−/−^ DC abolishes their enhanced glycolytic activity and SRC. Finally, our data suggest a possible pathway via which TORC2^−/−^ DC display augmented TORC1 metabolic activity and through which enhanced integrin alpha IIb (Itga2b) and protein kinase 2 (Ptk2)/focal adhesion kinase (FAK) expression leads to increased hematopoetic cell signal transducer (Hcst; also known as PIK3AP) expression upstream of TORC1 activity, increased NFκβ activation, enhanced eukaryotic translation initiation factor 4E binding protein 1 (4EBP1), and subsequent increased nuclear receptor peroxisome proliferator-activated receptor gamma (Pparγ), sterol regulatory element-binding transcription factor 1 (Srebf1) and CD36 (fatty acid translocase) expression downstream of TORC1 activity. Our findings suggest that TORC2 may restrain TORC1-regulated metabolic function in myeloid DC.

## Materials and Methods

### Mice

C57BL/6 (B6) CD11c-CreRictor^f/f^ (herein referred to as TORC2^DC−/−^) mice were generated (35) by crossing B6 mice in which *rictor* is flanked by loxP restriction digest sites (generously provided by Drs. Keunwook Lee and Mark Boothby, Vanderbilt University School of Medicine) with B6 mice expressing Cre recombinase on the CD11c promoter (CD11c-Cre; The Jackson Laboratory). The genetic background of crossed mice was verified by polymerase chain reaction (PCR) genotyping; CD11c-Cre- littermates were used as negative controls.

### Generation and Stimulation of BM-Derived DC

Femoral BM cells were harvested and cultured as described (36) using mouse recombinant GM-CSF alone (1,000 U/mL; R&D Systems, Minneapolis, MN; CAA26822). On d 6 of culture, DC were purified using anti-CD11c immunomagnetic beads (Miltenyi Biotec, Bergisch, Germany). Where indicated, the TLR4 ligand LPS (100 ng/mL; *Salmonella minnesota* R595; Alexis Biochemicals, San Diego, CA; ALX-581-008) was used to stimulate the DC for 16–18 h.

### Metabolism Assays

A Seahorse XFe96 Bioanalyzer (Agilent, Santa Clara, CA) was utilized to measure metabolic flux in real-time. DC were plated on Cell-Tak-coated Seahorse culture plates (100,000 cells/well) in assay media consisting of minimal, unbuffered DMEM supplemented with 1% v/v BSA and 25 mM glucose, 1 mM pyruvate, and 2 mM glutamine. Basal extracellular acidification rate (ECAR) and oxygen consumption rate (OCR) were taken for 30 min. Cells were stimulated with oligomycin (2 μM), the potent mitochondrial oxidative phosphorylation uncoupler carbonyl cyanide 4 p-(trifluoromethoxy) phenylhydrazone (FCCP) that disrupts ATP synthesis (1 μM), 2-deoxyglucose (2-DG; 10 mM), and rotenone/antimycin A (rot/AA) (0.5 μM) to obtain maximal respiratory and control values. Where indicated, DC were cultured with rapamycin (10 ng/mL; LC Laboratories, Woburn, MA) for 18 h after CD11c^+^ immunomagnetic bead selection on culture day 6. Where indicated, DC were stimulated with LPS (100 ng/mL) added to the cultures for 18 h, as indicated above.

ATP concentrations were determined using an ATP determination kit (ThermoFisher, Waltham, MA) as per the manufacturer's instructions. Where indicated, DC were stimulated with LPS (100 ng/mL) for 1 h.

### Quantification of Mitochondrial (mt)DNA

Real-time quantitative PCR (q-PCR) was used to quantify mtDNA copy number (37). Total DNA was isolated using the DNeasy Blood & Tissue Kit (QIAGEN GmbH, Hilden, Germany), according to the manufacturer's instructions. Mitochondrially-encoded nicotinamide adenine dinucleotide NADH dehydrogenase 1 (mND1) and hexokinase gene 2 (HK2) DNA products were amplified as described below under Quantitative PCR. To quantify mtDNA copy number, the ratio of mt DNA(ND1) to nuclear DNA(HK2) was calculated using the ^ΔΔ^Ct method. Primers used for ND1 were forward: 5′-CTAGCAGAAACAAACCGGGC-3′ and reverse: 5′-CCGGCTGCGTATTCTACGTT-3′; for HK2 forward: 5′-GCCAGCCTCTCCTGATTTTAGTGT-3′ and reverse: 5′-GGGAACACAAAAGACCTCTTCTGG-3′.

### Flow Cytometric Analysis

Mitochondrial mass and membrane potential were assessed using MitoTracker® Green FM (0.1 μM; Cell Signaling Technology, Danvers, MA) and tetramethylrhodamine ethyl ester (TMRE; 0.05 μM, ThermoFisher), respectively, according to the manufacturers' instructions. To assess viability, cells were stained with 7-amino-actinomycin (7-AAD; (BioLegend, San Diego, CA) in accordance with the manufacturer's instructions. Data were acquired with a Fortessa flow cytometer (BD Biosciences, San Jose, CA) and analyzed using FlowJo (TreeStar, Ashland, OR).

### NanoString Analysis

Total RNA was extracted from bead-purified CD11c^+^ DC generated from the BM of Ctrl or TORC2^DC−/−^ mice using an RNeasy Mini Kit (Qiagen, Hilden, Germany) as per the manufacturer's instructions. NanoString analysis was performed using a Mouse Immunology Panel (NanoString Technologies, Seattle, WA) as described (38).

### Quantitative PCR

cDNA was amplified using Platinum Quantitative PCR SuperMix-UDG (Invitrogen, Waltham, MA) in 10 μl volumes in quadruplicate with gene-specific primers and probed on the ABI Prism 7900HT Sequence Detection System (Applied Biosystems, Foster City, CA) according to the manufacturer's instructions. Thermal cycling conditions were 50°C for 2 min then 95°C for 2 min, followed by 40 cycles of 95°C for 15 s and 60°C for 1 min. Data were analyzed using the ΔΔCt method with expression normalized to the housekeeping gene GAPDH.

### Western Blots

DC pellets were lysed in RIPA lysis buffer (Sigma-Aldrich) with Protease/Phosphatase Inhibitor Cocktail (Cell Signaling Technology). Proteins were then separated with SDS-PAGE 4–20% gel (20 μg protein/slot; Precast Gels, Genscript, Piscataway, NJ) and transferred onto 8.5 × 6 cm PVDF membranes (GE Healthcare, Freiburg, Germany) and blocked with 5% w/v BSA (0.5 h). Membranes were incubated overnight with primary Abs: rabbit anti-p-4E-BP1 (Thr37/46), rabbit anti-p-p70S6K (Thr389/412), rabbit anti-Pparγ, rabbit anti-p-NFκβ p65 (Cell Signaling Technology; 1:1000), rabbit anti-CD36 (Abcam, Cambridge, MA, 1:1000) or mouse anti-β-actin (Sigma-Aldrich; 1:1:2000). HRP-conjugated goat anti-rabbit IgG (H±L) secondary Ab (Cell Signaling Technology; 1:5000) was used (1 h). Signals were detected by Western HRP Substrate on ChemiDoc™MP Imaging System (Sigma-Aldrich; Bio-Rad, Hercules). Densitometric quantification of Western blot signals was performed using Image Lab software (open source: http://www.bio-rad.com/en-us/product/image-lab-software?ID=KRE6P5E8Z). All proteins were subsequently normalized to β-actin.

### Confocal Microscopy

Cell suspensions were fixed for 1 h in 2% v/v paraformaldehyde then cytospun (ThermoFisher CytoSpin 4) onto charged slides (Superfrost/Plus; ThermoFisher). The cells were permeabilized with 1% Triton X-100 in PBS for 15 min, blocked with 5% normal goat serum for 30 min then stained with primary antibody (Ab) directed against Trans Golgi Network 38 (rabbit anti-TGN38, Novus, Littleton, CO) (39), diluted 1:100 in PBS supplemented with 0.5% BSA (PBB). Cells were washed x4 with PBS and stained with secondary goat anti-rabbit Alexa 488 (ThermoFisher; 1:500), rhodamine Phalloidin (F-actin, ThermoFisher) and 0.1% Hoechst's dye (nuclei; ThermoFisher). Confocal images were obtained by the Center for Biological Imaging, University of Pittsburgh, on a Nikon A1 microscope using 100 x objective and zoomed using 1.24 Nyquist. Maximum intensity projections were 3-D constructed and analyzed using NIS Elements software (Nikon, Tokyo, Japan).

### Fluorescence Imaging and Quantitation of Lipid Droplets

Cell suspensions were fixed in 2% paraformaldehyde for 20 min at room temperature, washed with PBS and permeabilized in 0.1% saponin (Sigma-Aldrich, St. Louis, MO) for 10 min. The cells were then stained for 45 min with HCS LipidTox Red neutral lipid stain (Invitrogen), diluted 200-fold in PBS, according to manufacturer's instructions. Subsequently, nuclear staining was performed using 4′, 6-diamidino-2-phenylindole (DAPI; Sigma-Aldrich). Confocal images were obtained as described above. Lipid droplets were counted manually, yielding each time data from 60 cells using NIS Elements software (Nikon, Tokyo, Japan).

### Transmission Electron Microscopy

Cell suspensions were fixed in 2.5% v/v glutaraldehyde then pelleted immediately in a 1.5 ml microfuge tube at 300 x G. Pellets were then post-fixed for 1 h in 1% OsO_4_, 1% K_3_Fe(CN)_6_ and dehydrated through a graded series of 30–100% ethanol, 100% propylene oxide, then infiltrated in a 1:1 mixture of propylene oxide:Polybed 812 epoxy resin (Polysciences, Warrington, PA) for 1 h. After several changes of 100% resin over 24 h, the pellet was embedded in a final change of resin, cured at 37°C overnight, followed by additional hardening at 65°C for 2 more days. Ultrathin (70 nm) sections were collected on 200 mesh copper grids, stained with 2% uranyl acetate in 50% methanol for 10 min, followed by 1% lead citrate for 7 min. Sections were imaged using a JEOL JEM 1400 transmission electron microscope (Peabody, MA) at 80 kV fitted with a side mount AMT 2k digital camera (Advanced Microscopy Techniques, Danvers, MA).

### Statistical Analyses

Results are expressed as means ± 1SD. Significances of differences between groups were determined using Student's ‘*t*'-test or one-way ANOVA Tukey's multiple comparisons test (GraphPad Prism) as indicated, with *p* < 0.05 considered significant.

## Results

### TORC2^−/−^ DC Display Augmented Glycolytic Capacity, Glycolysis-Dependent ATP Production and Viability Compared to Ctrl DC

**Figure 1:**
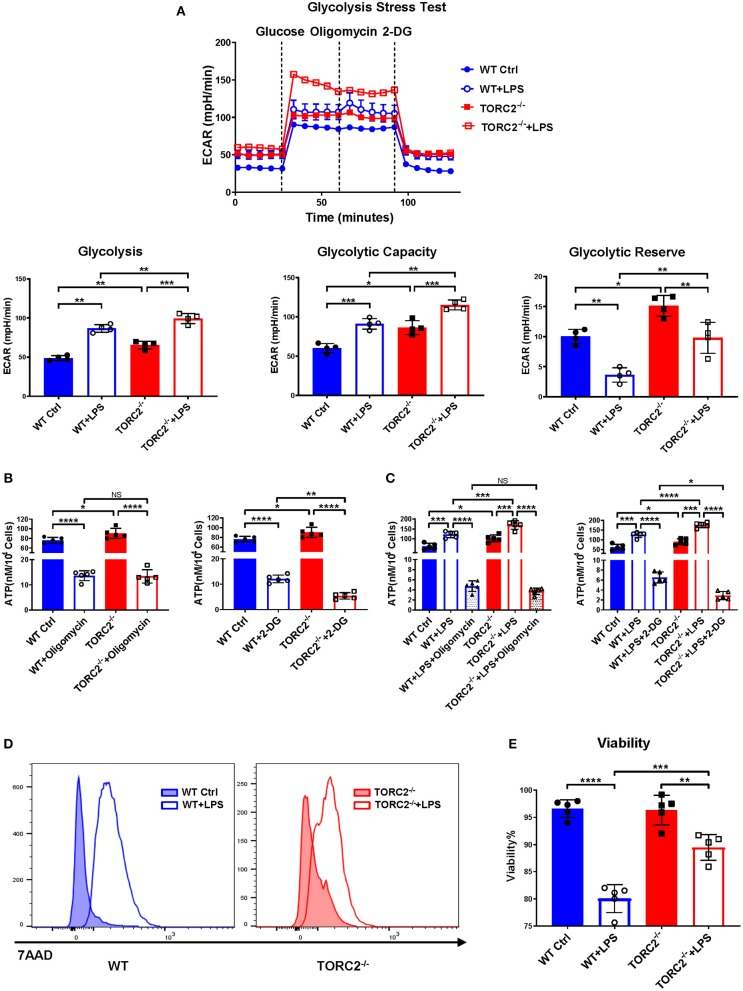
TORC2^−/−^ DC display augmented glycolytic activity, glycolysis-dependent ATP production and viability compared to wild-type (WT) control (Ctrl) DC. Bone marrow-derived DC were generated from WT C57BL/6 Ctrl or TORC2^DC−/−^ mice (TORC2^−/−^ DC), with or without LPS stimulation, and analyzed using a Seahorse XFe96 Bioanalyzer for metabolic flux in real-time over 125 min. **(A)** Representative glycolysis stress test showing basal glycolysis (ECAR), glycolytic capacity and glycolytic reserve; *n* = 4 independent experiments, with at least 2 mice per experiment; one-way ANOVA Tukey's multiple comparisons test, *p < 0.05, **p < 0.01, ***p < 0.001. **(B)** ATP production by non-stimulated WT Ctrl DC or TORC2^−/−^ DC. **(C)** ATP production by WT Ctrl DC or TORC2^−/−^ DC stimulated with LPS for 1 h; one-way ANOVA Tukey's multiple comparisons test, *p < 0.05, **p < 0.01, ***p < 0.001, ****p < 0.0001. **(D)** Representative histogram of 7AAD viability dye staining. **(E)** Quantification of viability as percentage of live (7AAD^−^) cells; **(B–E)** *n* = 5 independent experiments, with at least 2 mice per experiment; one-way ANOVA Tukey's multiple comparisons test, **p < 0.01, ***p < 0.001, ****p < 0.0001. ATP, adenosine triphosphate; 2-DG, 2-deoxyglucose; ECAR, extracellular acidification rate; NS, not significant.

To investigate the impact of TORC2 deletion on DC metabolic function, we assessed glycolysis via extracellular flux, as measured by basal extracellular acidification rate (ECAR). Glycolysis was elevated significantly in TORC2^−/−^ compared with WT control (Ctrl) DC and in both Ctrl DC and TORC2^−/−^ DC following LPS stimulation (Figure 1A). Interestingly, glycolytic capacity was also increased significantly in non-stimulated TORC2^−/−^ DC compared to Ctrl DC, and in LPS-stimulated TORC2^−/−^ DC compared to Ctrl DC. Glycolytic reserve was higher in TORC2^−/−^ DC, both without and with LPS stimulation. While there was no significant difference in ATP production between Ctrl and TORC2^−/−^ DC following inhibition of OXPHOS with oligomycin, Ctrl and TORC2^−/−^ DC displayed significantly decreased ATP production when glycolysis was inhibited with 2-DG (Figure 1B; non-stimulated; Figure 1C + LPS 1 h). TORC2^−/−^ DC stimulated with LPS also had significantly higher viability than Ctrl DC stimulated with LPS (representative histogram Figure 1D; quantified in Figure 1E); however, the immediate glycolytic response to LPS stimulation did not different significantly between Ctrl DC and TORC2^−/−^ DC (representative ECAR Supplementary Figure 1A; quantified in Supplementary Figure 1B).

### TORC2^−/−^ DC Exhibit Increased Spare Respiratory Capacity (SRC), Mitochondrial Biomass, Mean Mitochondrial DNA Copy Number and Mitochondria That Fail to Depolarize Following LPS Stimulation

**Figure 2:**
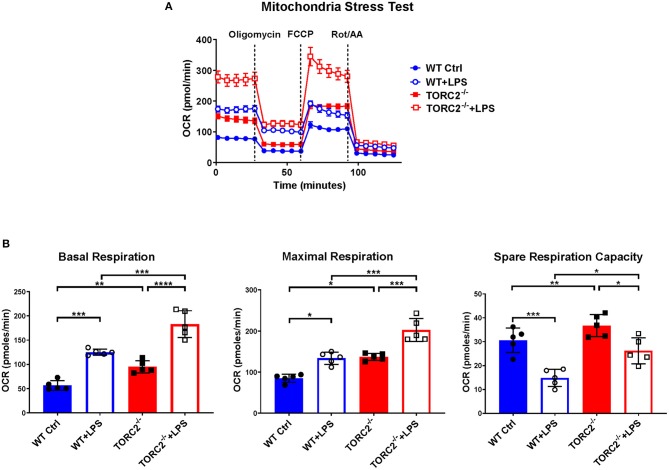
TORC2^−/−^ DC exhibit enhanced respiration and spare respiratory capacity (SRC). Bone marrow-derived DC were generated from WT control (Ctrl) or TORC2^DC−/−^ mice (TORC2^−/−^ DC), then stimulated with LPS for 18 h, as indicated. The DC were analyzed using a Seahorse XFe96 Bioanalyzer for metabolic flux in real-time over 125 min with (1) oligomycin, (2) FCCP, and (3) Rot/AA injected at the times indicated to obtain control values. **(A)** Representative mitochondria stress test of non-stimulated or LPS-stimulated WT Ctrl DC or TORC2^−/−^ DC. **(B)** Quantification of basal respiration, maximal respiration and spare respiratory capacity (SRC). SRC was calculated as the difference in OCR after addition of FCCP (2) and OCR before the addition of oligomycin (1); *n* = 5 independent experiments with at least 2 mice per experiment; one-way ANOVA Tukey's multiple comparisons test, *p < 0.05, **p < 0.01, ***p < 0.001, ****p < 0.0001. FCCP, carbonyl cyanide 4 p-(trifluoromethoxy) phenylhydrazone; Rot/AA, rotenone/antimycin A.

**Figure 3:**
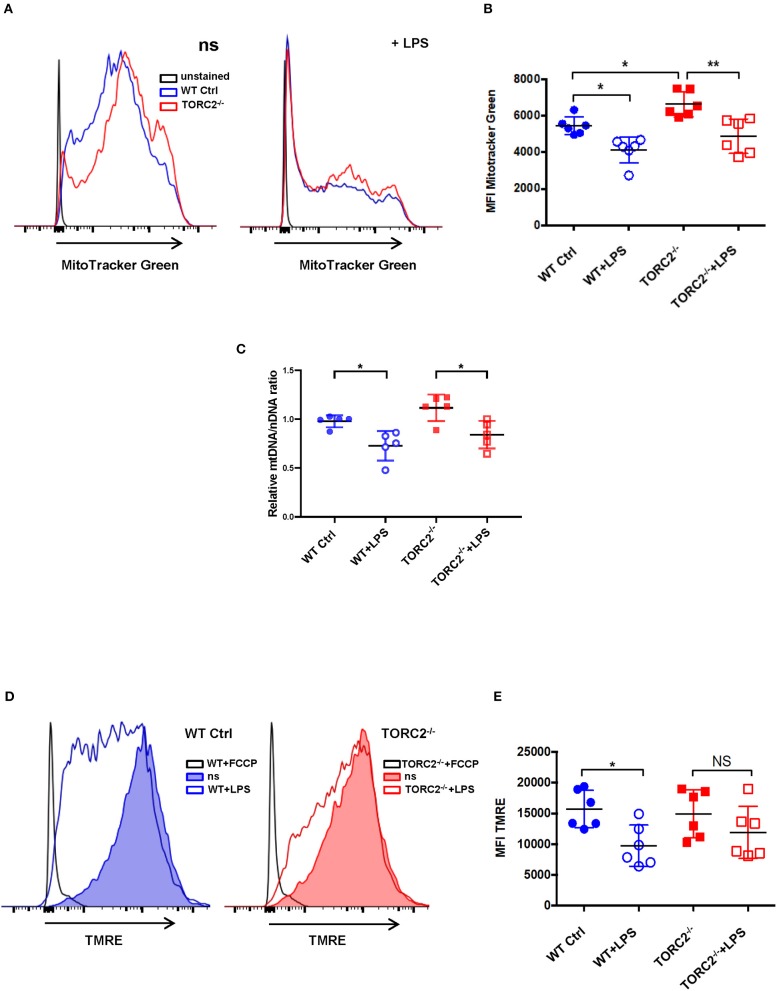
TORC2^−/−^ DC exhibit increased mitochondrial biomass and mitochondria that fail to depolarize following LPS stimulation. Bone marrow-derived DC were generated from wild-type (WT) control (Ctrl) or TORC2^DC−/−^ mice (TORC2^−/−^ DC), then stimulated or not with LPS for 18 h, as indicated. **(A)** Representative flow cytometry histograms of WT Ctrl and TORC2^−/−^ DC stained with MitoTracker Green. ns, non-stimulated. **(B)** Quantification of mean fluorescence intensity (MFI) of MitoTracker Green; *n* = 6 independent experiments with at least 2 mice per experiment; one-way ANOVA Tukey's multiple comparisons test, *p < 0.05, **p < 0.01. **(C)** Quantification of mitochondrial (mt)DNA copy number by measuring mtDNA relative to nuclear DNA using qPCR; **(D)** Representative flow cytometry histograms of Ctrl DC and TORC2^−/−^ DC stained with TMRE. **(E)** Quantification of MFI of TMRE; **(B–E)**, *n* = 5–6 independent experiments with at least 2 mice per experiment; one-way ANOVA Tukey's multiple comparisons test, *p < 0.05. NS, not significant; TMRE, tetramethylrhodamine ethyl ester.

SRC, calculated as the difference in oxygen consumption rate (OCR) measured via extracellular flux after addition of oligomycin and prior to addition of FCCP, was elevated significantly in non-stimulated TORC2^−/−^ DC compared to Ctrl DC, without or with LPS stimulation (representative OCR Figure 2A; quantified in Figure 2B). We next assessed how TORC2 deletion in DC might influence mitochondrial phenotype. Mitochondrial mass, as determined by fluorescent mitochondrial labeling (MitoTracker Green), was significantly greater in non-stimulated TORC2^−/−^ DC compared with non-stimulated Ctrl DC (representative histograms Figure 3A; quantified in Figure 3B) and decreased significantly in both cell populations following LPS stimulation (Figure 3B). Non-stimulated TORC2^−/−^ DC had a greater mean mt DNA copy number (although not statistically significant; Figure 3C) compared with Ctrl DC; mtDNA copy number decreased significantly in both Ctrl and TORC2^−/−^ DC following LPS stimulation (Figure 3C). It has been reported that macrophages stimulated with LPS undergo mitochondrial depolarization (40). However, TORC2^−/−^ DC mitochondria failed to depolarize significantly following LPS stimulation (as opposed to Ctrl DC that did), as assessed via uptake of cationic TMRE fluorescent dye (representative histograms Figure 3D; quantified in Figure 3E).

### TORC2^−/−^ DC Display More Compact Golgi Stacks With Less Perinuclear Localization Compared With Ctrl DC

**Figure 4:**
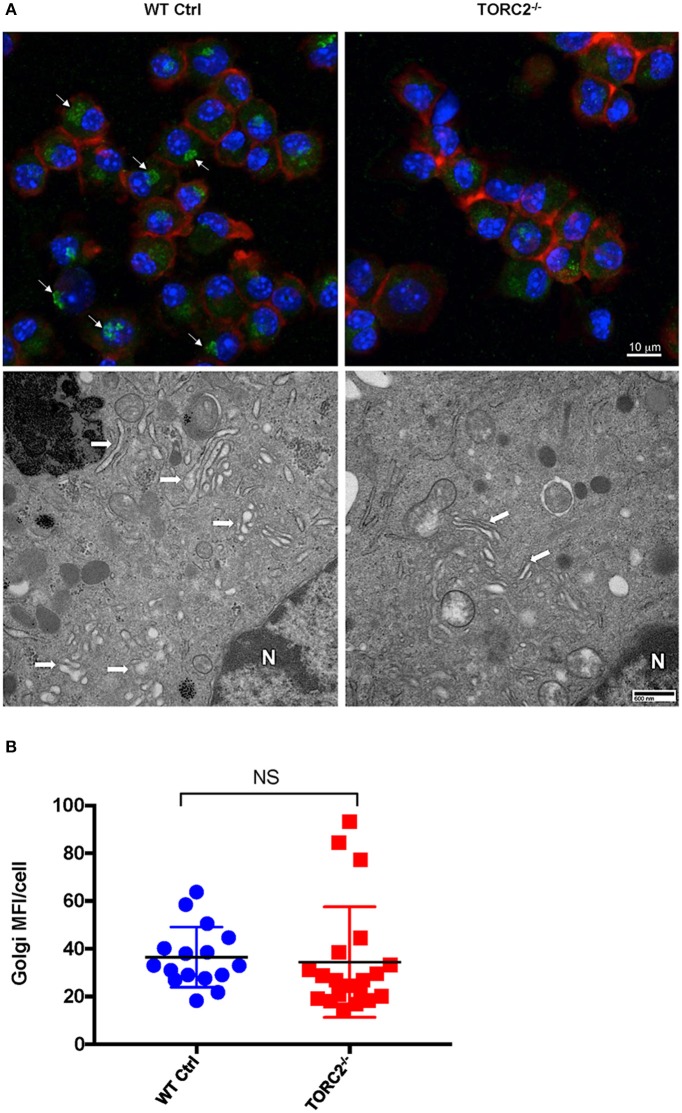
TORC2^−/−^ DC display more compact Golgi stacks with less perinuclear localization compared to wild-type (WT) control (Ctrl) DC. DC were generated from WT Ctrl or TORC2^DC−/−^ mice (TORC2^−/−^ DC) and prepared for confocal microscopy and transmission electron microscopy (TEM). **(A)** Representative maximum intensity projection of DC immuno-stained for Golgi (green), F-actin (red), and nuclei (blue), with arrows marking perinuclear Golgi (top panel); representative TEM of DC with arrows marking Golgi, and N marking the cell nucleus (bottom panel). **(B)** Quantification of total mean fluorescence intensity (MFI) in 3-dimensional images of Golgi stain per cell; each point represents values from one high power field (HPF); *n* = 4 mice per group; 4–5 HPFs per mouse. Student's “*t*”-test, NS, not significant.

It has previously been posited that the critical role of aerobic glycolysis in activated DC is to produce tricaboxylic acid cycle intermediates necessary for lipogenesis and subsequent Golgi apparatus and endoplasmic reticulum expansion to support *de novo* protein synthesis (30). As we observed enhanced glycolytic activity in TORC2^−/−^ DC, we next determined whether TORC2 deficiency in DC impacted the localization, structure, and quantity of Golgi apparatus. To determine if TORC2 deficiency in DC impacted the localization, structure and quantity of Golgi apparatus, we first immunostained DC with anti-TGN38 Ab and assessed the location of the Golgi relative to cell nuclei and plasma membrane. In Ctrl DC, the Golgi complex was perinuclear, whereas in TORC2^−/−^ DC the Golgi showed less intense staining and were dispersed throughout the cell (Figure 4A, top panel). We next used TEM to assess the structure of the Golgi. In Ctrl DC, the Golgi cisternae appeared more dilated, and more Golgi were observed perinuclearly; in contrast, the Golgi in TORC2^−/−^ DC appeared more compact, with less Golgi visible perinuclearly (Figure 4A, bottom panel). Finally, we quantified the MFI of TGN38 staining from 3-D stacks of confocal images to assess the total Golgi content of the DC. We observed no significant differences in total Golgi content as determined by TGN38 staining in Ctrl DC compared with TORC2^−/−^ DC, but localization was clearly affected (Figure 4B).

### Inhibition of TORC1 Activity in TORC2^−/−^ DC Leads to Loss of Their Enhanced SRC and Glycolytic Capacity

**Figure 5:**
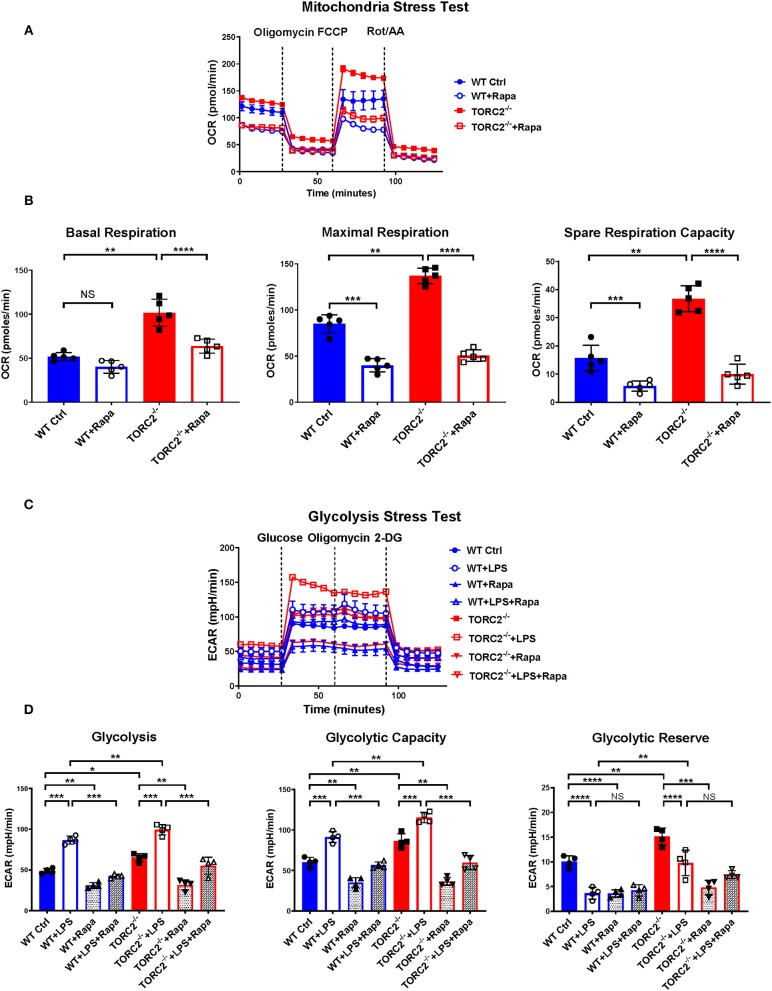
Inhibition of TORC1 activity in TORC2^−/−^ DC leads to loss of their enhanced spare respiratory capacity (SRC) and glycolytic capacity. DC were generated from wild-type (WT) control (Ctrl) or TORC2^DC−/−^ mice (TORC2^−/−^ DC), then cultured with or without low concentration rapamycin (Rapa) (10 ng/mL) for 18 h. DC were analyzed using a Seahorse XFe96 Bioanalyzer for metabolic flux in real-time over 125 min with (1) oligomycin, (2) FCCP, (3) Rot/AA injected at the times indicated to obtain control values. **(A)** Representative mitochondria stress test showing oxygen consumption rate (OCR). **(B)** Quantification of basal OCR, maximal OCR and spare respiratory capacity (SRC). SRC was calculated as the difference in OCR after addition of FCCP (2) and OCR before the addition of oligomycin (1); *n* = 5 independent experiments with at least 2 mice per experiment; one-way ANOVA Tukey's multiple comparisons test, **p < 0.01, ***p < 0.001, ****p < 0.0001. **(C)** Representative glycolysis stress test showing responses of Ctrl or TORC2^−/−^ DC cultured with or without rapamycin and stimulated or not with LPS for 18 h; **(D)** Quantification of basal glycolysis (ECAR), glycolytic capacity and glycolytic reserve; *n* = 4 independent experiments with at least 2 mice per experiment; one-way ANOVA Tukey's multiple comparisons test, *p < 0.05; **p < 0.01; ***p < 0.001, ****p < 0.0001. 2-DG, 2 deoxyglucose; ECAR, extracellular acidification rate; FCCP, carbonyl cyanide 4 p-(trifluoromethoxy) phenylhydrazone; NS, not significant; Rot/AA, rotenone/antimycin A.

While the exact mechanisms remain unclear, it has been reported that TORC1 and TORC2 may exert some regulatory influence on each other, Akt and insulin-dependent PI3K signaling (41). Therefore, to elucidate whether TORC2 in DC had TORC1-independent or TORC1-dependent metabolic regulation, we incubated both Ctrl DC and TORC2^−/−^ DC with RAPA (10 ng/mL) for 18 h, then analyzed their SRC and glycolytic capacity via extracellular flux, as in Figures 1, 2. Inhibition of TORC1 in TORC2^−/−^ DC abolished the increase in SRC (representative OCR Figure 5A; quantified in Figure 5B) and glycolytic activity (representative ECAR Figure 5C; quantified in Figure 5D) in non-stimulated cells compared with Ctrl DC. Inhibition of TORC1 in both LPS-stimulated Ctrl DC and TORC2^−/−^ DC reduced their glycolytic activity significantly (Figures 5C,D).

### TORC2^−/−^ DC Exhibit a Distinct Gene Expression Profile From Ctrl DC

**Figure 6:**
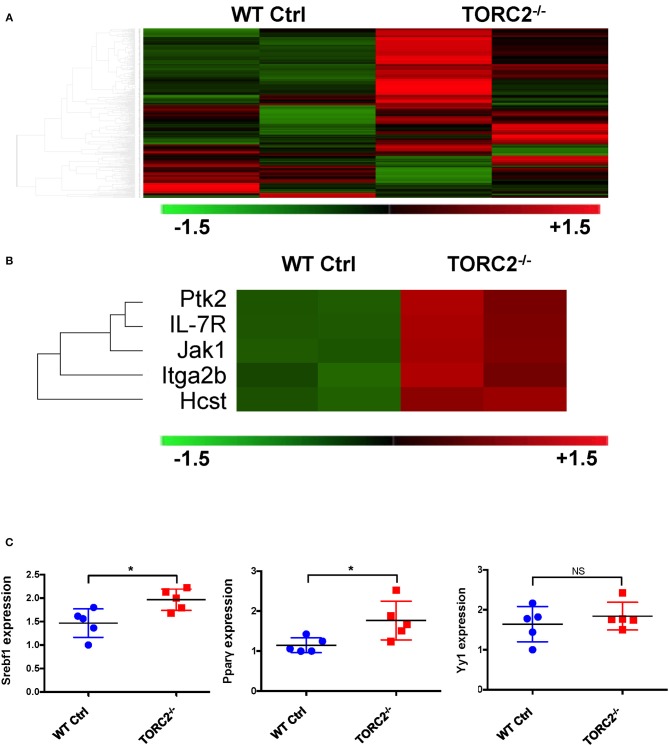
TORC2^−/−^ DC exhibit a distinct gene expression profile from wild-type (WT) control (Ctrl) DC. **(A)** Expression of 378 genes comprised in the NanoString Mouse Immunology Panel, shown as a heat map. Red indicates increased gene expression and green indicates decreased gene expression compared to Ctrl DC; *n* = 2 mice in each group. **(B)** Selection highlighting 5 genes of interest,- upstream mediators of augmented Akt/TORC1 activity from the panel in **(A)**, that were upregulated in TORC2^−/−^ compared with Ctrl DC. **(C)** Expression of Srebf1, Pparγ, and Yy1 mRNA determined by RT-PCR in WT Ctrl DC and TORC2^−/−^ DC and normalized to the housekeeping gene GAPDH, with Ctrl DC as the referent control; *n* = 5 mice per group, Student's “*t*”-test, *p < 0.05; NS, not significant.

To provide insight into possible mechanisms by which TORC2^−/−^ DC exhibit enhanced TORC1-dependent metabolic activity, we performed gene expression analysis to identify any differences in expression of genes between TORC2^−/−^ DC and Ctrl DC, as represented in the heat map in Figure 6A. Five genes with enhanced expression in TORC2^−/−^ DC (Ptk2, IL-7R, Jak1, Itga2b, and the PI3K component Hcst; Figure 6B) were identified as upstream mediators of augmented Akt/TORC1 activity. We then performed qPCR to determine the relative expression of the downstream targets of TORC1,- the transcription factor Srebf1, the nuclear receptor Pparγ (that regulates fatty acid storage and glucose metabolism) and the transcription factor Yin Yang 1 (Yy1) that impact cell metabolism (17, 18, 42) in TORC2^−/−^ and Ctrl DC. Both Srebf1 and Pparγ were expressed at significantly higher levels in TORC2^−/−^ DC compared to Ctrl DC (Figure 6C), suggesting a role for these transcription factors in the altered metabolic profile of TORC2^−/−^ DC.

### TORC2^−/−^ DC Exhibit Enhanced Lipid Content

**Figure 7:**
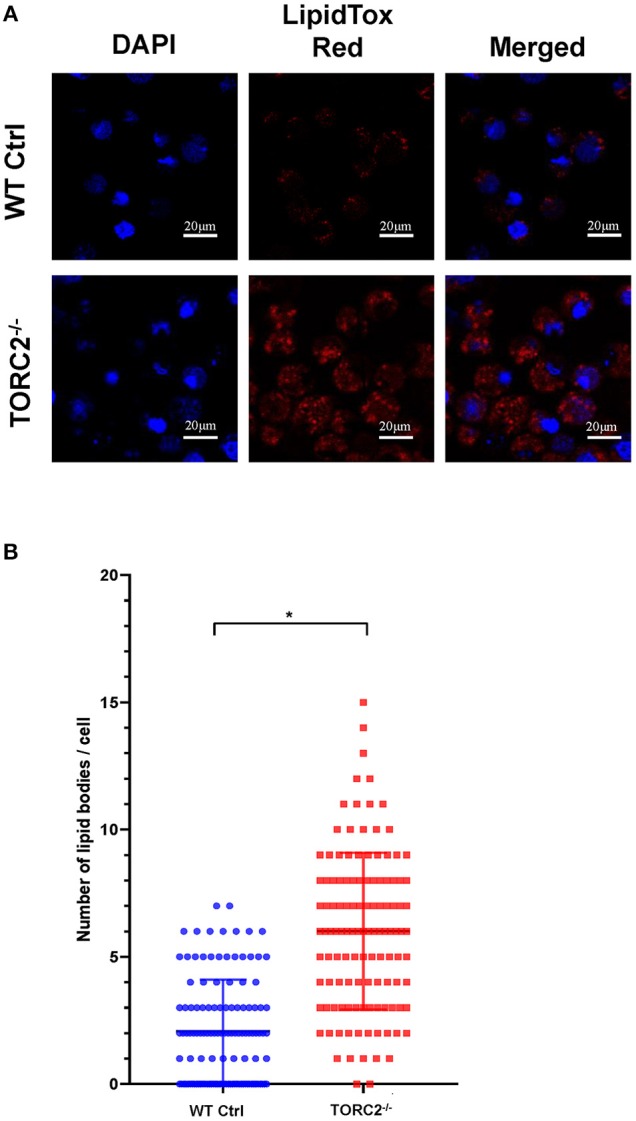
TORC2^−/−^ DC display more lipid droplets (LDs) compared to wild-type (WT) control (Ctrl) DC. DC were generated from WT Ctrl or TORC2^DC−/−^ mice (TORC2^−/−^ DC) and prepared for confocal microscopy. **(A)** LDs were stained with HCS LipidTox Red (red). **(B)** Quantification of the number of LDs per cell. Results of a representative experiment out of two performed is shown. Sixty cells were analyzed for each condition in each experiment; Student's “*t*”-test, *p < 0.05.

To ascertain the lipid content of TORC2^−/−^ DC, we stained lipid droplets (LDs) with HCS LipidTox Red neutral lipid stain. We found a significant increase in LDs in TORC2^−/−^ DC compared to Ctrl DC (Figures 7A,B).

### TORC2^−/−^ DC Display More Activated NFκβ Downstream of TORC1 Signaling

**Figure 8:**
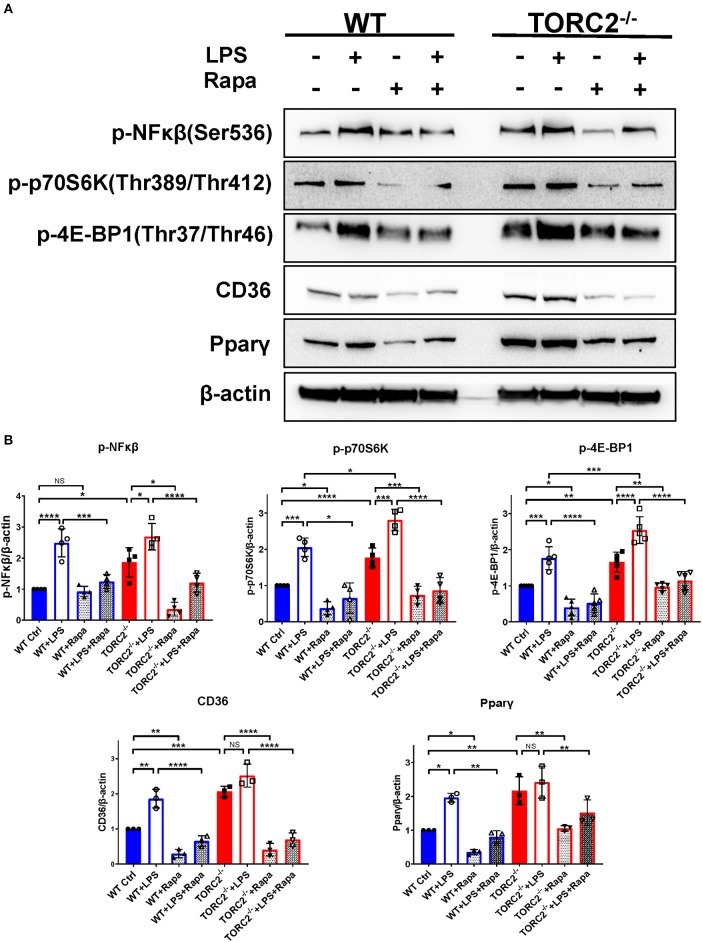
TORC2^−/−^ DC exhibit a protein expression pattern distinct from wild type (WT) control (Ctrl) DC. **(A)** Representative Western blots of p-NFκβ, p-P70S6K, p-4E-BP1, CD36 and Ppar-γ expression by unstimulated and LPS-stimulated (18 h) WT Ctrl or TORC2^−/−^ DC, in the presence or absence of rapamycin (Rapa). **(B)** Blots were quantified using Image-Lab software and normalized to β-actin (*n* = 3–4 independent experiments with at least 2 mice per experiment; *p < 0.05, **p < 0.01, ***p < 0.001, ****p < 0.0001). One-way ANOVA test Tukey's multiple comparisons test. NS, not significant.

To investigate NFκβ activation and the activation status of the TORC1 pathway, we performed Western blots to determine the expression of p-NFκβ (Ser536), p-p70S6K (Thr 389/Thr 412), downstream targets of TORC1 in both Ctrl DC and TORC2^−/−^ DC. As shown in Figures 8A,B, normalized p-NFκβ (Ser536), p-p70S6K (Thr 389/Thr 412), p-4EBP1(Thr37/Thr46), CD36, and Pparγ were augmented significantly in TORC2^−/−^ DC compared with Ctrl DC, with or without LPS stimulation. In the presence of rapamycin, a selective inhibitor of TORC1, the augmented TORC1 signaling activity observed in TORC2^−/−^ DC was reversed. Taken together, these findings suggest that TORC1 function is elevated in TORC2^−/−^ DC.

## Discussion

We and others have reported previously (13, 43) that murine myeloid DC lacking functional TORC2 display an augmented pro-inflammatory phenotype and enhance Th1/Th17 and CD8^+^ effector T cell responses *in vivo* (13–15). Similar observations have been reported for mouse macrophages (44). We now demonstrate that TORC2^−/−^ DC have an altered metabolic profile, whereby they exhibit enhanced glycolytic activity and spare respiratory capacity, dependence on glycolysis for ATP production, enhanced mitochondrial mass and lipid production, and increased viability following TLR4 stimulation. These changes may contribute to/support the enhanced stimulatory function reported for TORC2^−/−^ DC (13–15, 43).

Earlier studies have shown that while quiescent, immature DC have relatively low metabolic needs, these needs increase upon cell activation and maturation due to bioenergetic pressure to upregulate co-stimulatory molecule and pro-inflammatory cytokine production (29). The metabolic process that permits DC to meet these enhanced metabolic demands occurs via a “switch” from oxidative phosphorylation to aerobic glycolysis. There is evidence (33) that this initial “switch” toward increased glycolytic metabolism is not dependent on TOR signaling. However, TORC1 (but not TORC2) has been described as essential for DC glycolytic commitment (32). We found that in this study, immature (non-stimulated) myeloid DC lacking functional TORC2 (Rictor) had significantly increased glycolytic function compared to immature Ctrl DC, and that the increase in glycolytic capacity and reserve was also observed following stimulation of the DC with the TLR4 agonist LPS. These findings, together with our previous observation (13) of enhanced co-stimulatory CD86, together with decreased co-inhibitory programmed death ligand-1 (PD-L1) expression on TORC2^−/−^ DC compared with Ctrl DC, are consistent with an intermediate maturity phenotype and supported by the increased dependence of non-stimulated TORC2^−/−^ DC on glycolysis for ATP production that we observed.

In addition to its importance for nascent protein production, glycolytic commitment by mature DC is critical for their survival (32). Indeed, in conjunction with increased glycolytic activity, TORC2^−/−^ DC exhibited enhanced viability following exposure to LPS that can induce programmed cell death in DC (45). Thus, the enhanced pro-inflammatory function of TORC2^−/−^ DC that we have documented (13–15) *in vitro* and *in vivo* may also be attributed, in part, to their more robust viability. While T cells can trigger DC apoptosis through Fas and perforin as a mechanism to self-limit T cell activation (46, 47), such enhanced viability of activated TORC2^−/−^ DC may augment/extend their capacity to interact with and stimulate responder T cells.

In addition to augmented glycolytic activity, TORC2^−/−^ DC also displayed increased SRC, in conjunction with enhanced mitochondrial biomass, increased mean mitochondrial DNA, and mitochondrial failure to depolarize following LPS stimulation. Mitochondrial SRC has been described as the extra capacity of mitochondria to produce energy under increased cell stress and is correlated with prolonged cell survival and function (48). Given the enhanced viability of stimulated TORC2^−/−^ DC, an increase in SRC compared with LPS-stimulated Ctrl DC corroborates our findings. As CD8^+^ memory T cells have enhanced SRC due to increased mitochondrial biomass (48), the increased mitochondrial content of TORC2^−/−^ DC that we observed compared with Ctrl DC is consistent with our other findings. Failure of the mitochondrial membrane of TORC2^−/−^ DC to depolarize significantly following LPS stimulation was however surprising, as LPS has been shown to collapse mitochondrial membrane potential (ΔΨ_m_) in macrophages (40). On the other hand, there is evidence that, in macrophages, that disproportionately utilize ATP generated via glycolysis as protection against cell death (as we observed in TORC2^−/−^ DC), high ΔΨ_m_ is maintained via reverse functioning of F(o)F(1)-ATP synthase and adenine nucleotide translocase (40).

Enhanced glycolytic activity has been described as a means by which activated DC augment lipogenesis for increased production and transport of co-stimulatory molecules and cytokines (33). Indeed, we observed enhanced lipid production in the TORC2^−/−^ DC. We also assessed whether, in TORC2^−/−^ DC, there might be alterations in the localization, dilation and amount of Golgi apparatus that, in conjunction with the endoplasmic reticulum (ER), is critical for protein processing and transport. Surprisingly, given the enhanced glycolytic activity of TORC2^−/−^ DC, we did not observe any significant increase in the overall Golgi content of these cells. However, we did find that the Golgi of TORC2^−/−^ DC were less dilated than those in Ctrl DC, with less perinuclear localization. Recent studies (49) have favored a cisternal progenitor model of protein transport, whereby proteins are transported along tightly-compacted Golgi subcompartments that undergo fission and fusion events that allow stable protein transport to the membrane. Thus, the tightly compacted Golgi cisternae and diffuse TGN localization may reflect enhanced protein transport in these cells.

Given the mitochondrial dysregulation observed in TORC2^−/−^ DC, we investigated possible underlying mechanisms. Cells maintain mitochondrial homeostasis by controling both the number and quality of mitochondria via mitophagy (50). Interestingly, TORC1 is an important regulator of the nuclear transcription of genes necessary for mitochondrial biogenesis through peroxisome-proliferator-activated receptor coactivator-1α (PGC-1α) and yin-yang 1(YY1) (17). In addition, TORC1 regulates mitochondrial activity via phosphorylation of the mitochondrial membrane protein Bcl-X_L_ (51). TORC1 has also been reported to positively regulate glucose uptake (52) and mitophagy (53). As there is possible interplay between TORC1 and TORC2 through their interactions with Akt and the tuberosclerosis complexes 1/2 (TSC1/2), we assessed whether the mitochondrial dysregulation observed in TORC2^−/−^ DC could be attributed to compensatory augmented TORC1 activity. Indeed, as we show, inhibition of TORC1 with rapamycin abolished the enhanced glycolytic function and SRC of TORC2^−/−^ DC, suggesting that compensatory effects mediated through TORC1 might represent the key mechanism accounting for the enhanced mitochondrial activity and glycolytic function of TORC2^−/−^ DC.

In this study, we performed gene expression analysis that identified five genes of interest (upstream mediators of augmented Akt/TORC1 activity) that were differentially expressed between Ctrl DC and TORC2^−/−^ DC. These genes identified a signaling pathway in which the integrin subunit Itga2b, Ptk2 (also known as focal adhesion kinase; FAK) and Hcst (PIK3AP) are upregulated in TORC2^−/−^ DC. Integrin clustering has been shown to mediate intracellular signaling via the catalytic kinase Ptk2 (54), that leads ultimately to activation of the PI3K/Akt/TORC1 signaling pathway (55). Another possible mechanism for upregulation of TORC1 activity in TORC2^−/−^ DC based on these gene expression data, is upregulation of the IL-7R and Janus kinase 1 (JAK1), as IL-7R/JAK1 signaling can activate the PI3K signaling pathway (56).

**Figure 9:**
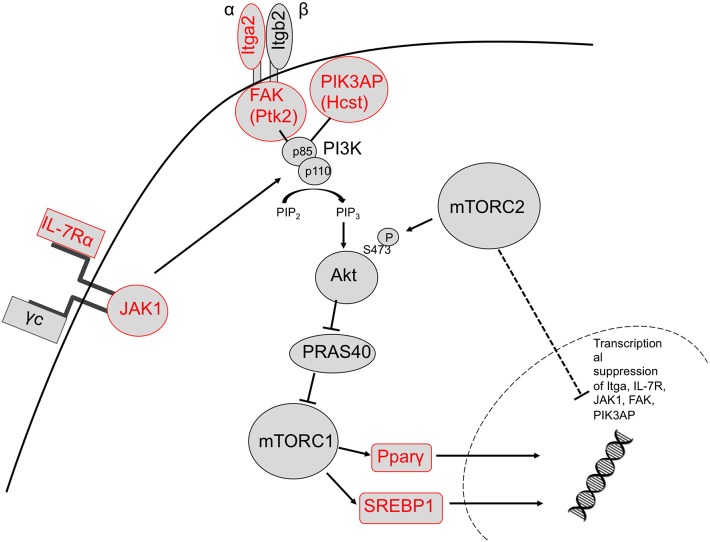
Proposed mechanism by which TORC2 may restrain TORC1 activity in DC via the transcriptional suppression of upstream TORC1 activators. Based on our observations, TORC2 activity may lead to transcriptional suppression of integrin (ItgA2 and FAK) and cytokine receptor (IL-7R) and JAK1 signaling in conjunction with PIK3AP upstream of Akt/mTORC1 activity, and ultimately the metabolic regulators SREBP1 and Pparγ downstream of TORC1 activity (upregulated genes labeled in red). Itga2b, integrin alpha II b; FAK, focal adhesion kinase (Ptk2, protein kinase 2), Hcst, hematopoietic cell signal transducer; Pparγ, peroxisome proliferator-activated receptor gamma; Sregf1, sterol regulatory element transcription factor 1.

We identified upregulation of two genes downstream of TORC1 signaling in TORC2^−/−^ DC that are known to regulate metabolic function: Pparγ and Srebf1. Activation of these transcription factors downstream of TORC1 leads to expression of lipogenic genes (57). It has been demonstrated (33) that glycolysis in DC drives lipogenesis and subsequent Golgi/ER expansion upon DC activation and more recently (42), that Pparγ signaling can also promote glycolysis in hematopoietic stem cells. Interestingly, loss of TORC2 function in yeast results in enhanced lipogenesis (58). Taken together, our data suggest that TORC2 may play a role in transcriptional control of TORC1-regulated metabolic processes, as outlined schematically in Figure 9.

Our findings suggest a novel role for mTORC2 in the negative regulation of DC metabolism. Its absence equips these APC to function with enhanced pro-inflammatory/T cell stimulatory activity, as we (13–15) and others have described (43). Myeloid DC lacking functional TORC2 are more glycolytically active and have increased dependence on glycolysis for ATP production. TORC2^−/−^ DC also have abnormalities in mitochondrial regulation, characterized by enhanced mitochondrial biomass and mitochondria that fail to depolarize following DC activation. The metabolic phenotype of TORC2^−/−^ DC is lost upon inhibition of TORC1, suggesting a significant compensatory effect of the mTORC1 pathway in TORC2^−/−^ DC. In addition, we have identified several genes upstream of TORC1, and the transcription factors Pparγ and Srebf1 downstream of TORC1, that are upregulated in TORC2^−/−^ DC. The data strongly suggest that TORC2 may function to restrain TORC1-driven anabolic metabolism in myeloid DC.

## Ethics Statement

All animal procedures were performed according to an Institutional Animal Care and Use Committee-approved protocol (#16058226) in accordance with National Institutes of Health (NIH) guidelines.

## Author Contributions

AW, HD, YZ, and RN performed experiments, analyzed that data, and wrote the manuscript. AG provided conceptual input and analyzed data. AM performed experiments. DS provided conceptual insights and supervised the cell imaging analyses. GD and AT provided conceptual insights and edited the manuscript. AT supervised the project. All authors provided feedback on the manuscript.

### Conflict of Interest Statement

The authors declare that the research was conducted in the absence of any commercial or financial relationships that could be construed as a potential conflict of interest.

## Abbreviations

Ctrl: control
DC: dendritic cell
2-DG: 2-deoxyglucose
4E-BP1: eukaryotic translation initiation factor 4E binding protein 1
ECAR: extracellular acidification rate
MFI: mean fluorescence intensity
OCR: oxygen consumption rate
Pparγ: peroxisome proliferator-activated receptor γ
SRC: spare respiratory capacity
Srebf1: sterol regulatory element transcription factor 1
(m)TOR(C): (mammalian) target of rapamycin (complex)
TMRE: tetramethylrhodamine ethyl ester.
