# Supplementary figures and images for: mTORC2 Deficiency Alters the Metabolic Profile of Conventional Dendritic Cells
Source: Front Immunol. 2019 Jul 2;10:1451. doi: 10.3389/fimmu.2019.01451 (PMC6626913; doi:10.3389/fimmu.2019.01451)

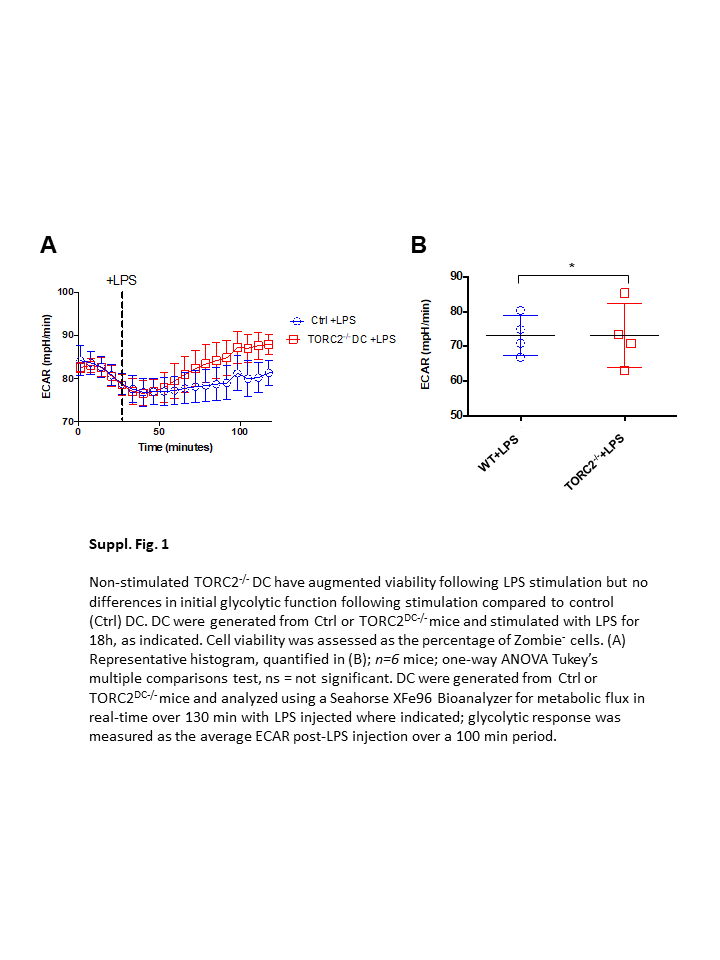

Supplement: Supplementary file 1 [file Image_1.TIF]
